# Place of death among adults with interstitial lung disease in the United States, 1999–2023: a national population-based study

**DOI:** 10.1097/MS9.0000000000004471

**Published:** 2025-12-03

**Authors:** Asad Ali Ahmed Cheema, Ali Salman, Fatima Raja, Hasan Akbar Naqvi, Komal Kumari, Fizza Mahmood, Ajwa Marrium, Muhammad Salar Khan Jadoon, Sanjana Devi, Ezza Bashir, Ibrahim Ahmed, Aizaz Afridi

**Affiliations:** a International School of Medicine, International University of Kyrgyzstan, Bishkek, Kyrgyzstan; b Department of Medicine, Dow University of Health Sciences, Karachi, Pakistan; c Department of Medicine, King Edward Medical University, Lahore, Pakistan; d Department of Medicine, Dow International Medical College, Karachi, Pakistan; e Department of Medicine, Islamabad Medical and Dental College, Islamabad, Pakistan; f Department of Medicine, Shifa College of Medicine, Islamabad, Pakistan; g Department of Medicine, Nawaz Sharif Medical College, Gujrat, Pakistan; h Department of Medicine, Indus Medical College, Tando Muhammad Khan, Pakistan; i Department of Medicine, Akhtar Saeed Medical and Dental College, Lahore, Pakistan; j Department of Medicine, Hayatabad Medical Complex, Peshawar, Pakistan

**Keywords:** end-of-life care, interstitial lung disease, mortality trends, palliative care, place of death

## Abstract

**Background:**

Interstitial lung disease (ILD) is a progressive disorder with high mortality and limited therapeutic options. National trends in the place of death among adults with ILD in the United States (U.S) remain poorly described, despite increasing emphasis on palliative and end-of-life care.

**Method:**

We analyzed U.S. mortality data (1999–2023) from the Centers for Disease Control and Prevention’s Wide-ranging Online Data for Epidemiologic Research (CDC WONDER) for adults aged ≥45 years with ILD. Deaths were categorized by location, and sociodemographic associations and temporal trends were assessed using multinomial logistic and Joinpoint regression.

**Result:**

A total of 388 120 ILD-related deaths occurred between 1999 and 2023. ILD mortality increased steadily over the study period, with an average annual percentage change of 2.75% (95% CI, 2.55–2.97; *P* < 0.000001). Nearly half occurred in hospitals (48.5%), followed by home (31.2%), hospice/nursing facilities (17.4%), and outpatient/emergency room (ER) (2.9%). The proportion of inpatient deaths declined from 60.7% in 1999 to 41.4% in 2023. In contrast, home deaths nearly doubled from 21.5% to 38.6%, while hospice/nursing facility deaths increased from 14.3% in 1999 to a peak of 19.5% in 2018, ending at 17.0% in 2023. Outpatient or ER deaths remained relatively stable throughout the study period. Adults ≥85 years most often died in hospice/nursing facilities, whereas those aged 75–84 years most often died in hospitals.

**Conclusion:**

Deaths in ILD have shifted away from hospitals toward home and hospice, while demographic disparities persist. These findings demand urgent end-of-life planning and equitable, targeted palliative care.

## Introduction

Interstitial lung diseases (ILDs) represent a group of over 200 chronic, progressive lung disorders that cause inflammation and scarring of the lung parenchyma. Among these, idiopathic pulmonary fibrosis (IPF) is the most prevalent and fatal subtype, primarily affecting older adults. IPF is associated with a median survival of just 3–5 years after diagnosis^[1,2]^. In the United States (U.S), an estimated 650 000 individuals are living with ILD, based on national prevalence data^[3]^. Despite the availability of antifibrotic therapies, most patients experience progressive deterioration, resulting in a significant physical and emotional burden, particularly during the terminal phase of the illness.

The place of death for patients with ILD has critical implications for end-of-life care quality. A randomized phase II trial of a home-based case-conference model (Hospital2Home) in advanced fibrotic ILD demonstrated significant short-term improvements in palliative symptoms and quality of life, supporting the importance of early palliative integration^[4]^. In contrast to oncology, where end-of-life care planning is better integrated, palliative care in ILD is often delayed or absent, mainly due to the disease’s unpredictable course and lack of early conversations about care preferences^[5]^. Analyzing patterns in the location of death provides valuable insight into how well the healthcare system supports patients in the final stages of ILD and helps identify gaps in aligning care with patient needs and preferences.

However, national data on where patients with ILD die and how this varies by demographics and geography remain sparse. Most large-scale end-of-life care studies focus on cancer or heart failure populations, leaving ILD underexplored despite its high mortality and symptom burden. Our study addresses this critical gap by examining ILD-related deaths in the U.S from 1999 to 2023, with a focus on trends and disparities in place of death. These findings aim to guide improvements in palliative access, care coordination, and overall quality of life in advanced ILD. This study complies with the TITAN 2025 Guidelines on Transparency in Artificial Intelligence Use in Research and Publications to ensure ethical and transparent reporting of AI use in manuscript^[6]^.

## Method

### Data source and study population

Mortality data were extracted from the Centers for Disease Control and Prevention’s Wide-ranging Online Data for Epidemiologic Research (CDC-WONDER) database, aggregating death certificate records across the U.S. We included all adult decedents aged 45 years and older who had ILD recorded as the underlying cause of death, based on the International Classification of Diseases, Tenth Revision (ICD-10) code J84 (Other interstitial pulmonary diseases). This encompasses subcategories such as J84.0 (alveolar and parietoalveolar conditions), J84.1 (other ILD with fibrosis), J84.8 (other specified ILD), and J84.9 (unspecified ILD). Due to database constraints, we could not reliably distinguish specific ILD subtypes (e.g., IPF, connective tissue disease–associated ILD, or hypersensitivity pneumonitis). The study period extended from 1999 to 2023. To reduce misclassification and maintain analytical consistency, we excluded deaths related to external causes such as unintentional injuries, suicide, homicide, or those under pending investigation. Reports lacking key demographic variables, such as sex, race, or location of death, were also excluded from analysis.

### Outcome measures

The primary outcome of interest was the place of death, as documented on the death certificate. The place of death was categorized into four categories: inpatient medical facility, outpatient facility or emergency department, nursing home or hospice, and the decedent’s home. This classification allowed for meaningful analysis of end-of-life care trends and enabled comparisons across care environments reflective of healthcare access, utilization, and patient or family preferences.

### Study variables

The following variables are included as covariates in the analysis: age group (45–54, 55–64, 65–74, 75–84, and >85 years), sex (men or women), race (White, Black, Asian or Pacific Islander), and ethnicity (Hispanic or Non-Hispanic). Geographic factors included U.S. Census Region (Northeast, Midwest, South, and West) and county-level urbanization status, defined using the National Center for Health Statistics Urban-Rural Classification Scheme. Urbanization categories were classified as large metropolitan, medium/ small metropolitan, or rural. These variables were chosen based on their association with healthcare delivery patterns and sociodemographics in end-of-life care.

**HIGHLIGHTS**

- Interstitial lung disease-related deaths in the United States rose to 388 000 over 25 years, with a steady 2.7% annual increase, underscoring its growing public health burden.
- Care-setting shifts: inpatient deaths declined from 61% to 41%, while home deaths nearly doubled, indicating a shift from hospital-centered care to settings such as home hospice, nursing facilities, or community palliative-care programs.
- Demographic trends: adults ≥85 years most often died in hospice or nursing facilities, while those 75–84 years most often died in hospitals.
- Disparities persisted: Black and Hispanic individuals, men, and residents of large metropolitan areas were less likely to die in supportive settings such as home or hospice.

### Statistical analysis

We used multinomial logistic regression to evaluate the association between demographic characteristics and place of death among adults with ILD. The dependent variable was the place of death, with an inpatient medical facility as the reference group. Independent variables included age group, sex, race, ethnicity, census region, and urbanization status. Age-adjusted mortality rates (AAMRs) were standardized to the 2000 U.S. population, and temporal trends were assessed using log-linear regression models. Joinpoint analysis was performed using the Joinpoint Regression Program (version 5.2.0, National Cancer Institute) and the Weighted Bayesian Information Criterion-guided model selection. Multicollinearity among covariates was assessed using variance inflation factors. The assumption of independence of irrelevant alternatives was evaluated using the Hausman-McFadden test. Odds ratios (ORs) with 95% confidence intervals (CIs) were calculated for each outcome category. A two-sided *P* value <0.05 was considered statistically significant. All statistical analyses were conducted using IBM SPSS Statistics, version 29.0 (IBM Corp., Armonk, NY).

## Results

### Annual mortality trend

Between 1999 and 2023, 388 120 deaths were attributed to ILD. Among the decedents, 359 217 (92.6%) were White individuals, 209 805 (54.1%) were men, and 146 495 (37.7%) were adults aged 75–84. Table 1 presents a summary of the aggregated data on the place of death stratified by demographics and clinical characteristics of decedents with ILD.

**Table 1:** Aggregated data for adults for places of death by decedent characteristics for interstitial lung disease (1999–2023)

	Total	Medical facility Inpatient	Medical facility – outpatient/ ER	Nursing/hospice	Decedent home
Decedent characteristics	*N* (%)	*N* (%)	*N* (%)	*N* (%)	*N* (%)
Total no. of deaths	388 120 (100%)	188 198 (48.5%)	11 138 (2.9%)	67 564 (17.4%)	121 220 (31.2%)
Gender					
Men	209 805 (54.1%)	105 604 (56.1%)	6790 (61%)	29 309 (43.4%)	68 102 (56.2%)
Women	178 315 (45.9%)	82 594 (43.9%)	4348 (39%)	38 255 (56.6%)	53 118 (43.8%)
Age groups					
45–54	11 005 (2.8%)	8364 (4.4%)	490 (4.4%)	339 (0.5%)	1812 (1.5%)
55–64	35 420 (9.1%)	24 312 (12.9%)	1268 (11.4%)	2249 (3.3%)	7591 (6.3%)
65–74	90 639 (23.4%)	51 632 (27.4%)	3174 (28.5%)	9122 (13.5%)	26 711 (22%)
75–84	146 495 (37.7%)	68 515 (36.4%)	4311 (38.7%)	24 210 (35.8%)	49 459 (40.8%)
85 +	104 561 (26.9%)	35 375 (18.8%)	1895 (17%)	31 644 (46.8%)	35 647 (29.4%)
Hispanic origin					
Hispanic	31 339 (8.1%)	17 812 (9.5%)	792 (7.1%)	2792 (4.1%)	9943 (8.2%)
Non-Hispanic	356 781 (91.9%)	170 386 (90.5%)	10 346 (92.9%)	64 772 (95.9%)	111 277 (91.8%)
Race					
Black	19 782 (5.1%)	13 336 (7.1%)	920 (8.3%)	1612 (2.4%)	3914 (3.2%)
White	359 217 (92.6%)	169 255 (89.9%)	10 066 (90.4%)	65 267 (96.6%)	114 629 (94.6%)
Asian	9121 (2.4%)	5607 (3%)	152 (1.4%)	685 (1%)	2677 (2.2%)
2013 Urbanization					
Large metro	189 791 (48.9%)	94 642 (50.3%)	5858 (52.6%)	30 556 (45.2%)	58 735 (48.5%)
Medium/small metro	129 524 (33.4%)	59 855 (31.8%)	3404 (30.6%)	24 497 (36.3%)	41 768 (34.5%)
Rural	68 805 (17.7%)	33 701 (17.9%)	1876 (16.8%)	12 511 (18.5%)	20 717 (17.1%)

ER, emergency room; metro, metropolitan area.

**Figure 1.**
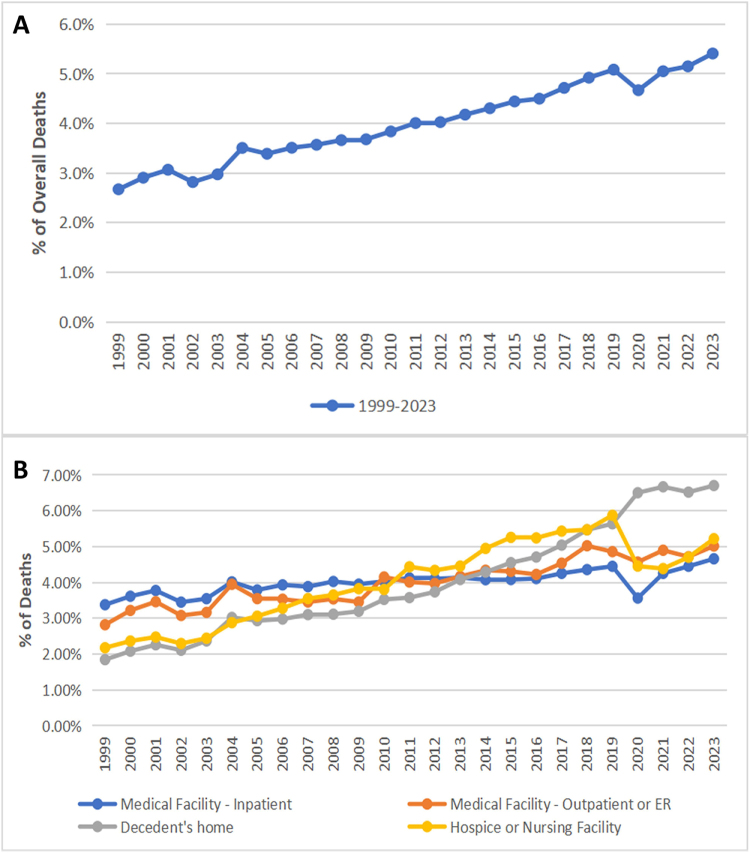
(A) Age-adjusted mortality rates for interstitial lung disease (ILD)-related deaths in the United States, 1999–2023. (B) Proportion of ILD-related deaths by place of death (inpatient medical facility, home, hospice/nursing facility, outpatient/emergency department), 1999–2023.

Yearly trend analysis revealed that ILD-related mortality increased steadily from 1999 to 2023, with an average annual percent change (AAPC) of 2.75 (95% CI, 2.55–2.97; *P* < 0.000001) (Fig. 1A).

There was a marked decrease in ILD deaths in inpatient medical facilities, from 60.7% (6404 by 10 534) in 1999 to 41.4% (8834 by 21 324) in 2023. The proportion of ILD deaths in outpatient or emergency settings remained relatively stable, from 3.4% (359 by 10 534) in 1999 to 2.9% (639 by 21 324) in 2023. In contrast, a significant increase was observed in the number of deaths at home, rising from 21.5% (2265 by 10 534) in 1999 to 38.6% (8237 by 21 324) in 2023. Similarly, the percentage of fatalities in hospice/nursing institutions increased from 14.3% (1506 by 10 534) in 1999 to 19.5% (3777 by 19 322) in 2018. This was followed by a decline to 15.2% (3037 by 19 917) in 2021 and a subsequent rise to 17.0% (3614 by 21 324) in 2023. The yearly trends in places of death attributable to ILD are shown in Fig. 1B.

**Figure 2.**
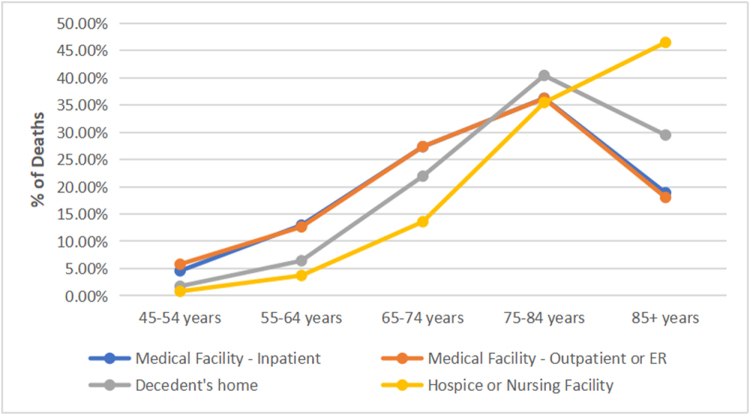
Place of death among decedents with interstitial lung disease by age group (45–54, 55–64, 65–74, 75–84, ≥ 85 years), United States, 1999–2023. ER, emergency room.

Adults aged 75–84 years accounted for the highest number of inpatient deaths, while individuals aged ≥85 years had the highest proportion of deaths occurring in hospice or nursing home settings (46.8%) (Fig. 2).

**Figure 3.**
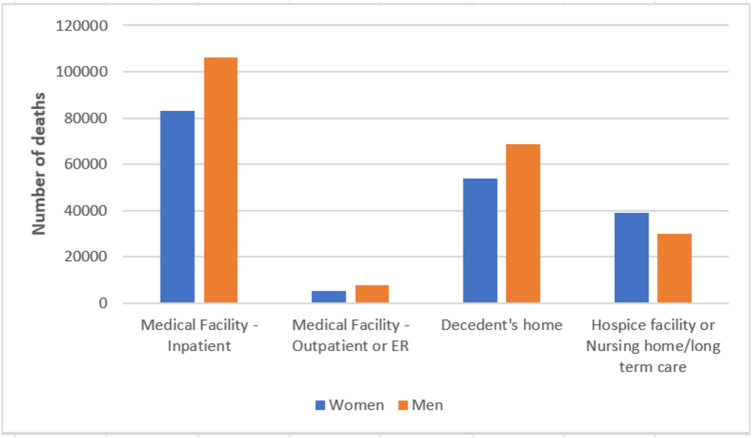
Adjusted odds ratios with 95% confidence intervals for place of death by sex among decedents with interstitial lung disease, using inpatient facility as the reference category. ER, emergency room.

#### Pre- and post-pandemic sensitivity analysis of ILD mortality and place of death

To further examine the effect of the coronavirus disease 2019 (COVID-19) era, mortality trends were stratified into pre-pandemic (1999–2019) and post-pandemic (2020–2023) periods using Joinpoint regression analysis. Overall ILD-related mortality increased steadily before the pandemic with an AAPC of 2.99% (95% CI, 2.75–3.23; *P* < 0.000001) (Supplemental Digital Content Figure S1, available at: http://links.lww.com/MS9/B46). After 2020, this rate accelerated significantly, with an AAPC of 4.67% (95% CI, 1.22–8.24; *P* = 0.028) (Supplemental Digital Content Figure S3, available at: http://links.lww.com/MS9/B46).

Inpatient deaths demonstrated only modest growth in the pre-pandemic years [AAPC 1.04% (95% CI, 0.80–1.28); *P* < 0.000001], but post-pandemic, they showed a sharper, though not statistically significant, rise [AAPC 8.57% (95% CI,−1.50–19.68); *P* = 0.068]. Deaths occurring in outpatient or emergency settings rose moderately pre-pandemic [AAPC 2.30% (95% CI, 1.84–2.77); *P* < 0.000001], whereas no statistically significant changes were observed post-pandemic [AAPC 2.45% (95% CI,−3.81–9.12); *P* = 0.24] (Supplemental Digital Content Figures S2 and S4, available at: http://links.lww.com/MS9/B46).

Home deaths displayed the strongest and most consistent increase before 2020, with an AAPC of 5.27% (95% CI, 4.87–5.68; *P* < 0.000001). However, this trend plateaued in the post-pandemic years, with no significant change (AAPC 0.69% (95% CI,−2.28–3.75]; *P* = 0.43). Hospice and nursing facility deaths also rose steadily before the pandemic (AAPC 5.20% (95% CI, 4.51–5.88]; *P* < 0.000001) but became unstable post-2020, with a non-significant increase [AAPC 5.72% (95% CI,—2.53–14.67]; *P* = 0.098) (Supplemental Digital Content Figures S2 and S4, available at: http://links.lww.com/MS9/B46).

This sensitivity analysis highlights that while pre-pandemic trends were steady and predictable, the post-pandemic period was characterized by accelerated overall mortality and more volatile patterns in place of death, suggesting a disruption of long-term trajectories due to healthcare system strain and shifts in care preferences during COVID-19.

### Sociodemographic predictors of death location in ILD

Multivariable logistic regression analysis identified significant associations between decedent characteristics and place of death. In the model, inpatient death was used as the reference category for location of death (Table 2).

**Table 2:** Multivariable logistic regression between decedent characteristics and place of death (1999–2023)

Categories	OR (95% CI)	P-vale
Origin		
Outpatient/ER		
Hispanic	0.712 (0.66–0.767)	<0.0001
Non-Hispanic	Reference	-
Race		
Blacks	2.684 (2.253–3.198)	<0.0001
White	2.37 (2.013–2.789)	<0.0001
Asian	Reference	-
Gender		
Men	1.214 (1.167–1.263)	<0.0001
Women	Reference	-
Age groups		
45–54	1.06 (0.956–1.175)	0.271
55–64	0.951 (0.884–1.024)	0.185
65–74	1.124 (1.06–1.192)	<0.0001
75–84	1.157 (1.094–1.223)	<0.0001
85 +	Reference	-
Urbanization		
Large metro	1.179 (1.117–1.245)	<0.0001
Medium/small metro	1.045 (0.986–1.107)	0.139
Rural	Reference	-
Nursing/hospice		
Origin		
Hispanic	0.43 (0.412–0.448)	<0.0001
Non-Hispanic	Reference	-
Race		
Blacks	1.471 (1.335–1.621)	<0.0001
White	3.396 (3.13–3.685)	<0.0001
Asian	Reference	-
Gender		
Men	0.652 (0.64–0.664)	<0.0001
Women	Reference	-
Age groups		
45–54	0.053 (0.048–0.059)	<0.0001
55–64	0.117 (0.112–0.122)	<0.0001
65–74	0.217 (0.211–0.223)	<0.0001
75–84	0.417 (0.408–0.426)	<0.0001
85 +	Reference	-
Urbanization		
Large Metro	0.966 (0.941–0.991)	0.008
Medium/Small metro	1.157 (1.126–1.188)	<0.0001
Rural	Reference	-
Descendants home		
Origin		
Hispanic	0.867 (0.844–0.89)	<0.0001
Non-Hispanic	Reference	-
Race		
Blacks	0.786 (0.741–0.834)	<0.0001
White	1.472 (1.404–1.544)	<0.0001
Asian	Reference	-
Gender		
Men	1.029 (1.014–1.044)	<0.0001
Women	Reference	-
Age groups		
45–54	0.236 (0.224–0.249)	<0.0001
55–64	0.325 (0.315–0.335)	<0.0001
65–74	0.523 (0.513–0.535)	<0.0001
75–84	0.72 (0.706–0.733)	<0.0001
85 +	Reference	-
Urbanization		
Large metro	1.067 (1.045–1.09)	<0.0001
Medium/small metro	1.159 (1.134–1.185)	<0.0001
Rural	Reference	-

ER, emergency room; metro, metropolitan area; and OR, odds ratio.

#### Age

Compared with adults aged ≥85 years, those aged 45–84 years had significantly lower odds of dying in hospice or nursing facilities. The lowest odds were observed among adults aged 45–54 years [OR, 0.05 (95% CI, 0.05–0.06)], followed by those aged 55–64 years [OR, 0.12 (95% CI, 0.11–0.12)], 65–74 years [OR, 0.22 (95% CI, 0.21–0.22)], and 75–84 years [OR, 0.42 (95% CI, 0.41–0.43)].

For outpatient or ER deaths, individuals aged 65–74 years [OR, 1.12 (95% CI, 1.06–1.19)] and 75–84 years [OR, 1.16 (95% CI, 1.09–1.22)] had modestly increased odds compared with the reference group. Adults aged 45–64 years did not demonstrate statistically significant differences in the odds of dying in these settings. For home deaths, adults aged 45–84 years consistently exhibited lower odds of dying at home than adults aged ≥85 years across all age strata.

#### Gender

Men had higher odds of dying in outpatient or emergency settings [OR, 1.21 (95% CI, 1.17–1.26)] compared with women. Conversely, they had lower odds of dying in hospice or nursing facilities [OR, 0.65 (95% CI, 0.64–0.66)]. For home deaths, men had slightly increased odds [OR, 1.07 (95% CI, 1.06–1.09)]. These results are illustrated in Figure 3.

#### Race and ethnicity

Black individuals had significantly higher odds of dying in outpatient or ER settings [OR, 2.68 (95% CI, 2.25–3.20)] and lower odds of dying at home [OR, 0.78 (95% CI, 0.74–0.83)], compared with Asian individuals. In contrast, White individuals had elevated odds of death in outpatient or ER settings [OR, 2.37 (95% CI, 2.01–2.79)] and hospice or nursing facilities [OR, 3.40 (95% CI, 3.13–3.69)] compared with Asian individuals. They also had significantly higher odds of home death [OR, 1.95 (95% CI, 1.80–2.12)].

Hispanic decedents had lower odds of dying in outpatient or ER settings [OR, 0.71 (95% CI, 0.66–0.77)] and hospice/nursing facilities [OR, 0.43 (95% CI, 0.41–0.45)] compared with non-Hispanic decedents. They also had modestly reduced odds of dying at home [OR, 0.91 (95% CI, 0.88–0.94)].

#### Urbanization

**Figure 4.**
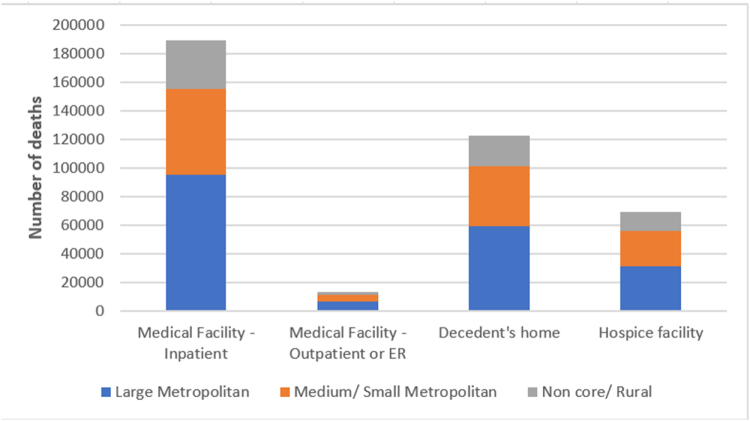
Adjusted odds ratios with 95% confidence intervals for place of death by urbanization category among decedents with interstitial lung disease, using inpatient facility as the reference category.

Compared with individuals in rural areas, those residing in medium or small metropolitan areas had increased odds of dying in hospice or nursing facilities [OR, 1.16 (95% CI, 1.13–1.19)]. In contrast, residents of large metropolitan areas had lower odds of death in these settings [OR, 0.97 (95% CI, 0.94–0.99)] and were more likely to die in inpatient settings [OR, 1.18 (95% CI, 1.12–1.25)]. No statistically significant differences were observed in home or outpatient/ER death by urbanization category after adjustment (Fig. 4).

## Discussion

This study provides a national perspective on 25-year trends in the place of death among adults with ILD in the U.S. From 1999 to 2020, ILD-related mortality steadily increased, accompanied by a notable shift in the location of death away from inpatient hospital settings and toward home, hospice, and nursing facilities. In contrast, deaths occurring in emergency departments or outpatient settings remained relatively uncommon and stable over time. Demographic factors significantly influenced the location of death. Adults aged >85 years were more likely to die in hospice or nursing homes. Men had a higher likelihood of dying in emergency or outpatient settings compared to women. Racial and ethnic disparities were also evident: Black and Hispanic individuals were generally less likely to die at home or in hospice care, while White individuals had higher odds of dying in supportive care environments. Additionally, urban-rural differences were noticeable, with residents of large metropolitan areas more likely to die in hospitals compared to those living in rural or smaller metropolitan regions.

Our study demonstrates a sustained rise in ILD-related mortality across the U.S from 1999 to 2023, highlighting a persistent and growing public health burden consistent with prior national reports. Meng *et al* described a continuous upward trajectory in AAMR for ILDs between 1979 and 2021, reporting an APC of 1.96% for men and 2.12% for women, reflecting a consistent rise over four decades^[7]^. Similarly, Soin *et al* reported rising ILD mortality (1999–2020), especially in U.S.–Mexico border counties, underscoring geographic and structural disparities in care^[8]^.

Several plausible factors may explain this continued increase. First, the aging U.S. population contributes to this significantly, as ILD primarily affects older adults and age remains a well-established risk factor for disease incidence and progression^[9]^. Second, environmental exposures such as wildfire smoke, air pollution, and tobacco use have been increasingly implicated in the pathogenesis and exacerbation of ILD, especially in vulnerable regions of the country.^[10–12]^ Third, advances in diagnostic tools, including high-resolution computed tomography (HRCT), transbronchial lung cryobiopsy (TBLC), genomic classifiers, and artificial intelligence–based imaging, have enhanced the accuracy and frequency of ILD detection^[13–15]^. While these innovations have improved early diagnosis and reduced misclassification, they may also have contributed to the observed rise in reported ILD-related deaths. However, direct evidence supporting this link is limited. Finally, the COVID-19 pandemic likely magnified this burden. Patients with ILD faced elevated risks of severe outcomes and mortality from SARS-CoV-2 owing to underlying pulmonary fibrosis and reduced respiratory reserve, as shown by Drake *et al*, while indirect effects such as delayed care and disrupted pulmonary rehabilitation further worsened outcomes^[16,17]^.

In parallel with the rising burden of ILD-related mortality, our study revealed a substantial decline in the proportion of deaths occurring in inpatient medical facilities between 1999 and 2023, suggesting a broader transformation in end-of-life care. Supporting this shift, Jeganathan *et al* reported a 23% reduction in ILD-related hospitalizations across the U.S. between 2008 and 2018 using the National Inpatient Sample, while in-hospital mortality rates remained stable^[18]^. Similarly, Ho *et al* analyzed national discharge data from 2006 to 2016 and documented declining hospitalization rates among patients with ILD, including IPF, reflecting a move away from inpatient settings, particularly among those with stable or gradually progressive disease. Seasonal variability and acute triggers such as pneumonia and respiratory failure were noted, while improvements in outpatient care including vaccination uptake, early antibiotic therapy, home-based monitoring, and expanded ambulatory services likely reinforced this shift^[19]^.

The decline in inpatient deaths may also reflect advances in diagnostics and therapeutics. Widespread adoption of high-resolution imaging and minimally invasive biopsy techniques has enhanced diagnostic accuracy^[20]^, while standardized care protocols have reduced complications and readmissions^[21]^. The introduction of antifibrotic therapies since 2014 has further improved disease control and survival, particularly in IPF populations^[22]^. Finally, the COVID-19 pandemic likely accelerated the transition away from hospital-based care, as concerns about hospital-acquired infection and strained health systems prompted greater reliance on community-based and ambulatory management, even during acute illness episodes.

Following the decline in inpatient deaths, our study documents a substantial increase in the proportion of ILD-related deaths occurring at home between 1999 and 2023, mirroring broader national patterns in end-of-life care across chronic illnesses. This finding is consistent with literature showing that many patients with advanced respiratory disease strongly prefer to die at home, emphasizing autonomy, comfort, and the presence of family. Skorstengaard *et al* reported that patients with advanced lung disease overwhelmingly favored home as their preferred place of care and death^[23]^. While this trend may reflect progress toward more patient-centered care, it also raises significant concerns about whether sufficient palliative infrastructure is available to support such transitions. Patients with ILD are less likely to receive hospice services or adequate symptom management than those with lung cancer, contributing to poorer perceived quality of dying^[24]^. Referral rates to specialist palliative care remain low often delayed or absent due to prognostic uncertainty and fragmented systems^[25]^. Even when palliative services are provided, evidence suggests that current models may not adequately improve quality of life and in some cases may worsen outcomes^[26]^. Furthermore, studies from Canada and the United Kingdom highlight gaps in home-based palliative infrastructure, including limited access to coordinated care planning, optimized oxygen or ventilatory support, and effective symptom control^[27]^. Together, these findings underscore that while the increase in home deaths may align with patient preferences, it also reflects systemic deficiencies in delivering equitable and high-quality end-of-life care for ILD.

Several additional factors likely contributed to the rise in home deaths. The expansion of hospice and palliative care programs, along with their integration into community-based care, has facilitated greater opportunities for patients to remain at home during advanced stages of disease. The COVID-19 pandemic may have further accelerated this trend by intensifying patient and family preferences to avoid hospitals due to infection risks and strained healthcare systems^[28]^. At the same time, the rapid adoption of telemedicine provided a new avenue to deliver clinical support in the home setting. This modality may continue to reinforce this transition in the post-pandemic era^[29]^. However, not all home deaths reflect intentional patient choice, some may have resulted from unmet acute care needs or barriers to emergency services during public health crises^[28]^. Thus, while the increasing proportion of home deaths signals progress toward aligning care with patient values, it also exposes persistent gaps in palliative resources and highlights the urgent need to strengthen community-based care models for this vulnerable population.

Building on these trends in care settings, our study found that demographic characteristics, particularly age, played a significant role in determining the place of death among patients with ILD. Stratification by age revealed distinct patterns: hospice and nursing home deaths were most frequent among adults aged 85 and older, and least common among those aged 45–54. These age-related disparities align with broader trends in palliative care utilization. Historically, hospice has been underutilized among patients with ILD, as noted by Lindell *et al*, who found that only a minority of individuals with IPF, a primary ILD subtype, received hospice care near the end of life^[30]^. Our findings may reflect a gradual shift in this trend, with increased hospice use being particularly notable among the oldest patients with ILD. Cagle *et al* reported that individuals aged 85 and above were significantly more likely to receive hospice services compared to younger decedents^[31]^. Robison *et al* further confirmed this pattern, showing over threefold greater hospice use among adults aged 85 and older compared to those under 65, while adults aged 45–64 had substantially lower odds of hospice utilization^[32]^. These results support our observation that ILD patients aged 85 and above are increasingly receiving care in supportive settings, whereas those aged 45–54 remain underrepresented in hospice and nursing homes.

Following the age-related disparities, our analysis revealed significant differences in place of death by race and ethnicity. Black individuals were markedly less likely to die at home or in hospice settings, highlighting persistent systemic inequities in end-of-life care. These findings are consistent with prior literature. Johnson *et al* reported that Black decedents were more likely to die in hospitals and less likely to receive care at home, even after accounting for socioeconomic status and clinical factors^[33]^. Such disparities are often linked to limited access to high-quality home-based care, fewer informal caregiving resources, and deep-rooted mistrust of the healthcare system. Smith *et al* further identified cultural values, inadequate clinician-patient communication, and structural barriers such as housing instability^[34]^. Robison *et al* also demonstrated that Black individuals had significantly lower rates of hospice utilization and higher odds of institutional death compared to White counterparts^[32]^. Washington *et al* emphasized how religious beliefs, mistrust of medical institutions, and the lack of racial concordance in palliative care contribute to reduced engagement with home- and community-based end-of-life services^[35]^. These converging factors underscore the need for culturally tailored interventions and equitable access to palliative care across racial and ethnic groups.

In addition to age and racial differences, our study identified evident sex-based disparities in place of death among individuals with ILD. Men were more likely to die in outpatient or emergency care settings and significantly less likely to die in hospice or nursing facilities. These trends may reflect underlying sociocultural dynamics that shape care preferences and access to care. Prior literature suggests that masculine norms such as reluctance to express vulnerability, emphasis on independence, and resistance to perceived loss of control may delay or deter engagement with palliative care services.^[36–38]^ Plys *et al* emphasized that institutional care settings like hospice may conflict with identity constructs rooted in military or traditionally masculine roles, posing additional challenges to end-of-life transitions^[38]^. Saito *et al* further demonstrated that men tend to enter hospice later and have shorter stays, patterns associated with poorer outcomes^[39]^. In contrast, women are more likely to engage with palliative services earlier and adhere to end-of-life goals, which may account for their greater representation in hospice and nursing home settings observed in our study^[40]^.

Geographic location also emerged as an important determinant of end-of-life care among ILD decedents. In our study, individuals residing in large metropolitan areas were more likely to die in emergency or outpatient settings and less likely to die in hospice or nursing facilities compared to those in rural regions. These findings align with national data showing lower utilization of hospice services in densely urbanized areas^[41]^. In metropolitan settings, multiple systemic and logistical barriers, such as housing instability, fragmented care coordination, delayed referrals, and limited community-based palliative care infrastructure, may restrict timely access to supportive care^[37,42]^. Conversely, despite challenges such as workforce shortages and limited healthcare resources, rural populations may benefit from stronger continuity of care, earlier hospice engagement, and robust informal support networks, often facilitated through community familiarity and nurse-led services^[43,44]^. Addressing these urban-rural disparities will require targeted expansion of hospice delivery models in urban centers, greater investment in home-based palliative care, and enhanced provider and patient education to ensure equitable end-of-life experiences across geographic contexts.

## Study limitations

This study has several important limitations. First, mortality data were obtained from the CDC WONDER system, which relies on death certificates filled out by attending physicians or medical residents. These certificates may be prone to misclassification of both the underlying cause and the place of death, especially in complex multisystem illnesses such as ILD. Second, aggregated, de-identified data limit our ability to analyze individual-level clinical variables such as disease severity, comorbidities, treatments received, and patient or family preferences, all of which play a major role in shaping end-of-life trajectories but are not captured here. This restricts the interpretability of specific associations and may obscure nuanced contributors to variation in in-depth settings. Third, while we evaluated trends across demographic and geographic strata, the CDC database does not permit direct examination of the causal pathways underlying differences in mortality patterns across race, sex, or region. Fourth, due to systematic data suppression by the CDC for small subgroups, we were unable to include American Indian and Alaska Native individuals in our race-based analyses. This exclusion limits the inclusion of certain racial and ethnic groups in our analysis, and therefore, our findings may not be generalizable to these populations. Lastly, the study design precluded differentiation by ILD subtype, and excluding individuals under age 45 may limit generalizability to younger adults. Importantly, the place of death should not be interpreted as a proxy for the quality of end-of-life care. While dying at home or in hospice may align with patient preferences, it can also reflect unmet needs, and our dataset cannot capture these nuances.

## Conclusion

In this national, population-based study of 388 120 ILD-related deaths from 1999 to 2023, we observed a substantial decline in inpatient deaths, a doubling of home deaths, and fluctuating trends in hospice or nursing home utilization. Our findings also highlight that adults aged 75–84 years, men, Black individuals, and urban residents have lower access to supportive care settings. As ILD mortality continues to rise, there is an urgent need to strengthen equitable, patient-centered palliative and hospice care. Efforts should focus on policy reform, workforce and infrastructure expansion, culturally tailored interventions, and integration of telehealth to support goal-concordant care delivery.

## Data Availability

All data used in this study are publicly available through the CDC-WONDER platform https://wonder.cdc.gov/ and are included within the article.

## Research registration unique identifying number (UIN)

Not applicable – This study is a retrospective analysis of de-identified, publicly available mortality data from the CDC-WONDER database. It did not involve the recruitment of human participants and therefore did not require registration in a clinical trial or systematic review registry.

## Peer and provenance statement

This article was not commissioned and underwent external peer review.
